# Irreversibility analysis through neural networking of the hybrid nanofluid for the solar collector optimization

**DOI:** 10.1038/s41598-023-40519-5

**Published:** 2023-08-16

**Authors:** Sayer Obaid Alharbi, Taza Gul, Ilyas Khan, Mohd Shakir Khan, Saleh Alzahrani

**Affiliations:** 1 Mathematics Department, College of Science Al-Zulfi, Majmaah University, 11952 Majmaah, Saudi Arabia; 2 Department of Mathematics, City University of Science and Information Technology, Peshawar, 25000 Pakistan; 3 Department of Physics, College of Science Al-Zulfi, Majmaah University, 11952 Majmaah, Saudi Arabia; 4 Department of Mathematics, University College in Al-Qunfudhah, Umm Al-Qura University, Al-Qunfudhah, Saudi Arabia

**Keywords:** Mathematics and computing, Nanoscience and technology

## Abstract

Advanced techniques are used to enhance the efficiency of the energy assets and maximize the appliance efficiency of the main resources. In this view, in this study, the focus is paid to the solar collector to cover thermal radiation through optimization and enhance the performance of the solar panel. Hybrid nanofluids (HNFs) consist of a base liquid glycol (C_3_H_8_O_2_) in which nanoparticles of copper (Cu) and aluminum oxide (Al_2_O_3_) are doped as fillers. The flow of the stagnation point is considered in the presence of the Riga plate. The state of the solar thermal system is termed viva stagnation to control the additional heating through the flow variation in the collector loop. The inclusion of entropy generation and Bejan number formation are primarily conceived under the influence of physical parameters for energy optimization. The computational analysis is carried out utilizing the control volume finite element method (CVFEM), and *Runge–Kutta 4* (RK-4) methods. (FEATool Multiphysics) software has been used to find the solution through (CVFEM). The results are further validated through a machine learning neural networking procedure, wherein the heat transfer rate is greatly upgraded with a variation of the nanoparticle's volume fraction. We expect this improvement to progress the stability of heat transfer in the solar power system.

## Introduction

Energy is a need of our daily life and all our requirements are not possible without these energy resources. But, to meet the rapid demand for energy, greater resources are necessary and not possible everywhere. Therefore, the researchers focused on easily accessible and inexpensive energy resources. Solar energy is the advance and main development in this area and can cover most of the energy requirements. The transfer of solar energy in the other form of energy-based technologies depends on concentrated solar power (CSP), and photovoltaic. Photovoltaics has worked to convert solar energy into electricity, while CSP is more advanced to alter solar energy into thermal energy and electricity at the same time. (CSP) operate in the absence of sunlight and more efficiently in those areas where sunlight is not available at all times. The researchers observed through the experiments that the advanced nanomaterials are more prominent to improve the efficiency of the solar collector, heat exchangers based on the uniform particle distribution^1–3^, tools for the solubilization of the nanoparticles^4^, and optical absorption^5,6^. The optimization in the heat transfer is also a key factor in the energy sector and the researchers have used their efforts to predict the suitable materials for the enhancement of heat transfer. Also, ocean, wind, and geothermal are not green energy sources and solar is the cleanest source of energy. Managing stagnation and overheating situations without the risk of system failure and the need for maintenance work. This is not only true for solar thermal collectors, but also for implementation as a whole. Hiemenz^7^ was the first to introduce the idea of stagnation into the fluid flow by applying the similarity of variables concept. In particular, the flow from the stagnation point is very stable for the mass deposition and thermal transport rate^8^. The idea of stagnation is further extended by Gowda et al.^9^ in the flow of dust particles with the combination of Marangoni convection. Jamaludin et al.^10^ studied the relevant phenomena of stagnation point flow with the combination of suction and radiations. Nasir et al.^11,12^ have studied the stagnation point flow considering the riga plate and nanofluids. including the elucidation of the Radiative-Hiemenz flow over a stretched Riga plate in a hybridized nanofluid. Hybrid nanofluids are twisted by the synthesis of two nanoparticles (that include carbon nanotubes, non-metallic, metallic or metallic oxides, or a mixture of different sorts). Various studies synthesized hybrid nanocomposites using different nanoparticles. This research shows hybrid nanofluids relating to their theoretical study, thermophysical properties, solar radiation, thermal applications, and numerical estimation including machine learning. he available literature indicates that hybrid nanofluids are more effective than nanofluids in enhancing the thermal performance of base fluids as described in^13–18^. This is because the thermal performance of nanofluids is limited concerning their specific thermal and chemical properties. Hybrid nanofluids are most important for improving thermal performance based on the combination of various thermal and chemical properties. In the existing literature, most researchers have imposed the outer magnetic field on the flow of conductive fluids. For low-conductive fluids, the external magnetic field is insufficient to produce the required amount of current. To solve this problem, the researchers^19^ introduce the idea of adjusting the Lorentz force in parallel directions of the wall. This appliance is introduced with the name of Riga-plate in the form of an electromagnetic actuator, including flashing electrodes and stable magnets^20^. The Riga plate tends to control the separation of the boundary layer and reduce turbulence generation, which is the basic requirement for the solar panel. The use of the Riga plate for various types of fluid flow may be seen in^21–23^.

This study concentrated on the overview of subjects related to stagnation and heat in general, and in particular to solar thermal applications^24^. The main themes of the study are as follows. The outcomes of this look might be helpful to use the stagnation point flow idea for stabilizing the impact of heat consumption. The stagnation point flow in the case of the Riga plate and entropy generation with a kind of flow setup is not addressed so far. These terminologies are strongly effective to optimize the solar collector. Further, the proposed model is solved by RK-4 and CVFEM methodologies. Neural networking is imposed to validate the obtained results.

### Objectives

The recent work is novel based on the following points.

- Irreversibility analysis is used in the present work (Means entropy andBejan number idea) is used, while in our published work no such ideas were used.
- (C3H8O2) glycol-based (Cu-Al_2_O_3_ ) hybrid nanofluids are used.
- The natural conviction in the present work is nonlinear.
- The variable viscosity and variable thermal conductivity, model^7,12^, is extended for the (HNFs).

## Formulation

**Figure 1:**
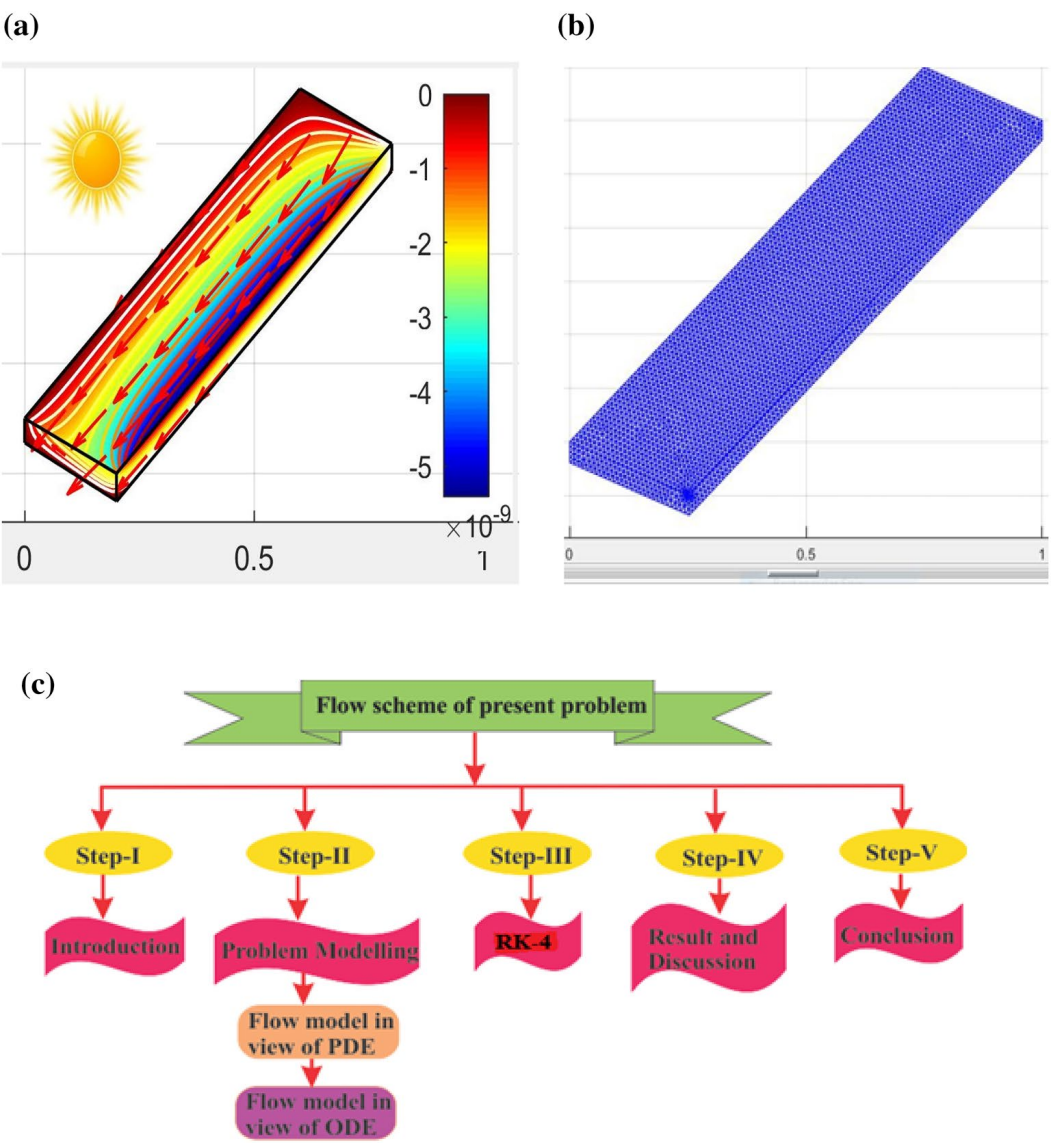
(**a**) Geometry (**b**) grids (**c**) flow chart.

In this formulation, we focused on the stagnation point flow of the (C_2_H_6_O_2_) hybrid nanofluids containing Cu and Al_2_O_3_ nanoparticles (NPs) as floculates. The Riga plate is used in the model problem which stabilizes the stagnation point flow. The stretching velocity is denoted by $u_{w} = \frac{{b\left( {x - x_{0} } \right)}}{{t_{ref} - \beta t}}$. A typical flow pattern is shown in Fig. 1a–c including panel structure and stagnation grids. The adjustment of the Riga plate is planned in such a way as to improve the thermal performance of the proposed model in terms of the internal Lorentz force and to reduce the turbulence effect during the flow pattern.

### Assumptions and model constraints

The assumptions are imposed based on the following points.

- The Cu and Al_2_O_3_ nanoparticles are in a stable relationship with the base fluids.
- The Lorentz force is used to reduce the turbulence behavior of the flow field and improve the thermal field.
- The flow is also presumed time-dependent.
- T_w_, and T_∞_ are used as the wall and free stream temperatures.

Based on the above discussion, all pertinent suppositions are used as indicated in the existing literature^7–14,18^. Core equations are demonstrated as.

1 $$\frac{\partial \,u}{{\partial \,x}} + \frac{\partial \,v}{{\partial \,y}} = 0,$$

2 $$\begin{aligned} \rho_{hnf} \left( {\frac{\partial u}{{\partial t}} + u\frac{\partial u}{{\partial x}} + v\frac{\partial u}{{\partial y}}} \right) & = \mu_{hnf} \left( {\frac{{\partial^{2} u}}{{\partial y^{2} }} + \frac{{\partial u_{e} }}{\partial t} + u_{e} \frac{{\partial u_{e} }}{\partial x}} \right) + \left( {\frac{{\pi j_{0} M_{0} }}{8}} \right)e^{{\left( {\frac{{ - \pi y_{1} }}{p}} \right)}} \hfill \\ & \quad + g\left( {\beta_{T} \rho } \right)_{hnf} \left( {T - T_{\infty } } \right) + g\left( {\beta_{T}^{2} \rho } \right)_{hnf} \left( {T - T_{\infty } } \right)^{2} , \hfill \\ \end{aligned}$$

3 $$\frac{\partial T}{{\partial t}} + u\frac{\partial T}{{\partial x}} + v\frac{\partial T}{{\partial y}} = \frac{{k_{hnf} }}{{\left( {\rho c_{p} } \right)_{hnf} }}\left( {\frac{{\partial^{2} T}}{{\partial y^{2} }}} \right) + \frac{{\mu_{nf} }}{{\left( {\rho cp} \right)_{nf} }}\left( {\frac{\partial u}{{\partial y}}} \right)^{2} - \frac{1}{{\left( {\rho c_{p} } \right)_{hnf} }}\left( {\frac{\partial q}{{\partial y}}} \right).$$

The initial and boundary conditions for the model problem are displayed as.

4 $$\begin{aligned} u(0) &= u_{w} ,v(0)\,\, = \,0,\,\,T(0) = T_{w} , \hfill \\ u(\infty ) &= u_{e} \,,T(\infty ) = T_{\infty } \,. \hfill \\ \end{aligned}$$

The velocity component along the x-direction is president as $\left( u \right)$, the velocity component along the y-direction is $(v),$ and the velocity term indicating the stagnation is $\left[ {u_{e} = \frac{{\alpha \left( {x - x_{0} } \right)}}{{t_{ref} - \beta t}}} \right]$. The subscripts $(nf),(hnf), \, (f)$ are used to represent the nanofluids, hybrid nanofluids, and base fluids, while $\mu ,\rho {,}\sigma , \, cp{, }k,$ indicate the dynamic viscosity, density, electrical conductivity, specific heat, and thermal conductivity, respectively.

The solar thermal radiations are obtained from the indicated form is:

5 $$q = - \frac{4}{3}\frac{{\sigma^{*} }}{{k^{*} }}\frac{{\partial T^{4} }}{\partial y},$$

### Alterations

The similarity variable which is used for alterations are:

6 $$\left[ {u,v,\eta ,\Theta ,} \right] = \left[ {\frac{{\alpha \left( {x - x_{0} } \right)}}{{t_{ref} - \beta t}}f^{\prime}(\eta ), - \alpha \sqrt {\frac{{\upsilon_{f} }}{{t_{ref} - \beta t}}} f(\eta ),\frac{y}{{\sqrt {\upsilon_{f} \left( {t_{ref} - \beta t} \right)} }}{, }\frac{{T - T_{\infty } }}{{T_{w} - T_{\infty } }},} \right].$$

7 $$\begin{aligned} f^{\prime\prime\prime} + \frac{{\rho_{hnf} }}{{\rho_{f} }}\frac{{\mu_{f} }}{{\mu_{hnf} }}\alpha \left[ {ff^{\prime\prime} - \left( {f^{\prime}} \right)^{2} + 1 - S\left( {\frac{\eta }{2}f^{\prime\prime} + f^{\prime} - 1} \right)} \right] \hfill \\ \quad + \frac{{\mu_{f} }}{{\mu_{hnf} }}MH\exp ( - \Lambda \eta ) + Gr\Theta + Gr^{*} \Theta^{2} = 0, \hfill \\ \end{aligned}$$

8 $$\left( {\frac{{k_{hnf} }}{{k_{f} }} + \frac{4}{3}Rd} \right)\Theta^{\prime\prime} + \Pr \frac{{(\rho cp)_{hnf} }}{{(\rho cp)_{f} }}\left[ {\alpha f\Theta^{\prime} - \frac{S\eta }{2}\Theta^{\prime}} \right] + \frac{{\mu_{f} }}{{\mu_{hnf} }}Ec\left( {f^{\prime}} \right)^{2} = 0,$$

The transform physical conditions are displayed as:

9 $$f^{\prime}(0) = c,f(0) = 0,\,f^{\prime}(\infty ) = 1,\,\Theta (\infty ) = 0,\Theta (0) = 1.$$

The embedded parameters that are obtained from the proposed alteration is.

10 $$\begin{aligned} Rd & = \frac{{4\sigma^{*} T_{\infty }^{3} }}{{k^{*} k_{f} }},\Pr = \frac{\mu Cp}{{k_{f} }},S = \frac{\beta }{\alpha },Ec = \frac{{u_{w}^{2} }}{{\left( {C_{p} } \right)_{f} (T_{w} - T_{\infty } )}}, \hfill \\ Gr &= \frac{{g\left( {\beta_{T} } \right)\left( {T_{w} - T_{\infty } } \right)x^{3} }}{{\upsilon_{f}^{2} }},Gr^{*} = \frac{{g\left( {\beta_{T}^{2} } \right)\left( {T_{w} - T_{\infty } } \right)^{2} x^{3} }}{{\upsilon_{f}^{2} }}, \hfill \\ MH & = \frac{{\pi j_{0} M_{0} }}{{8\alpha \rho_{f} }},\Lambda = \frac{{\pi \sqrt {\upsilon_{f} } }}{p},c = \frac{b}{\alpha }, \hfill \\ \end{aligned}$$

where $\alpha > 0,$ is a constant rate.

### Thermo-physical characteristics

Adding further for $\phi_{\text{Cu}} = \phi_{1} ,\,\,\phi_{{{\text{Al}}_{2} {\text{O}}_{3} }} = \phi_{2} ,$ signifying the volume concentration of $Cu\,\& \,Al2O3$. The characteristics of the hybrid nanofluids are displayed according to Table 1 and the same as^24^). These relations are the mathematical expressions to demonstrate the thermal properties of the hybrid nanoparticles.

11 $$\begin{aligned} \frac{{\rho_{hnf} }}{{\rho_{f} }} & = \left( {1 - \phi_{\text{Cu}} } \right)\left[ {\left( {1 - \phi_{{{\text{Al}}_{2} {\text{O}}_{3} }} } \right) + \phi_{{{\text{Al}}_{2} {\text{O}}_{3} }} \frac{{\rho_{{{\text{Al}}_{2} {\text{O}}_{3} }} }}{{\rho_{f} }}} \right] + \phi_{\text{Cu}} \frac{{\rho_{\text{Cu}} }}{{\rho_{f} }},\frac{{\mu_{hnf} }}{{\mu_{f} }} = \frac{1}{{\left( {1 - \phi_{{{\text{Al}}_{2} {\text{O}}_{3} }} } \right)^{2.5} \left( {1 - \phi_{\text{Cu}} } \right)^{2.5} }}, \hfill \\ \frac{{(\rho cp)_{hnf} }}{{\left( {\rho cp} \right)_{f} }} & = \left( {1 - \phi_{\text{Cu}} } \right)\left[ {\left( {1 - \phi_{{{\text{Al}}_{2} {\text{O}}_{3} }} } \right) + \phi_{{{\text{Al}}_{2} {\text{O}}_{3} }} \frac{{\left( {\rho cp} \right)_{{{\text{Al}}_{2} {\text{O}}_{3} }} }}{{\left( {\rho cp} \right)_{f} }}} \right] + \phi_{\text{Cu}} \frac{{\left( {\rho cp} \right)_{\text{Cu}} }}{{\left( {\rho cp} \right)_{f} }}, \hfill \\ \frac{{\sigma_{hnf} }}{{\sigma_{nf} }} & = \frac{{\left( {1 + 2\phi_{{{\text{Al}}_{2} {\text{O}}_{3} }} } \right)\sigma_{{{\text{Al}}_{2} {\text{O}}_{3} }} + \left( {1 - 2\phi_{1} } \right)\sigma_{nf} }}{{\left( {1 - \phi_{{{\text{Al}}_{2} {\text{O}}_{3} }} } \right)\sigma_{\text{Cu}} + \left( {1 + \phi_{{{\text{Al}}_{2} {\text{O}}_{3} }} } \right)\sigma_{nf} }},\frac{{\sigma_{nf} }}{{\sigma_{f} }} = \frac{{\sigma_{\text{Cu}} \left( {2\phi_{\text{Cu}} + 1} \right) + \sigma_{f} \left( {1 - 2\phi_{\text{Cu}} } \right)}}{{\sigma_{\text{Cu}} \left( {1 - \phi_{\text{Cu}} } \right) + \sigma_{f} \left( {\phi_{\text{Cu}} + 1} \right)}}, \hfill \\ \frac{{k_{hnf} }}{{k_{nf} }} & = \left( {\frac{{k_{{{\text{Al}}_{2} {\text{O}}_{3} }} + 2k_{nf} - 2\phi_{{{\text{Al}}_{2} {\text{O}}_{3} }} \left( {k_{nf} - k_{{{\text{Al}}_{2} {\text{O}}_{3} }} } \right)}}{{k_{{{\text{Al}}_{2} {\text{O}}_{3} }} + 2k_{nf} + \phi_{{{\text{Al}}_{2} {\text{O}}_{3} }} \left( {k_{nf} - k_{{{\text{Al}}_{2} {\text{O}}_{3} }} } \right)}}} \right),\frac{{k_{nf} }}{{k_{f} }} = \left( {\frac{{k_{\text{Cu}} + 2k_{f} - 2\phi_{\text{Cu}} \left( {k_{f} - k_{\text{Cu}} } \right)}}{{k_{\text{Cu}} + 2k_{f} + \phi_{\text{Cu}} \left( {k_{f} - k_{\text{Cu}} } \right)}}} \right), \hfill \\ \end{aligned}$$

**Table 1:** Thermophysical properties are presented as^24^.

Physical characteristics	Al_2_O_3_	C_3_H_8_O_2_	Cu
$k\;\left( {{\text{W}}/{\text{mK}}} \right)$	40	34.5	400
$c_{p} \;\left( {{\text{J}}/{\text{kg}}\,{\text{K}}} \right)$	765	4338	385
$\rho \;\left( {{\text{kg}}/{\text{m}}^{3} } \right)$	3970	5060	8933
$\beta \left( {1/k} \right)$	0.85	0.00062	1.67
$\sigma \;\left( {{\text{s}}/{\text{m}}} \right)$	35 × 10^6^	0.5 × 10^6^	59.6 × 10^6^

### Cu-Al_2_O_3_/C_3_H_8_O_2_ nanofluid simulations

The extended thermophysical characteristics of $\mu_{hnf}$ and $k_{hnf}$ are used as in^7,25^:

12 $$\begin{gathered} \frac{{\mu_{hnf} }}{{\mu_{f} }} = (306\phi_{\text{Cu}}^{2} - 0.19\phi_{\text{Cu}} + 1)(306\phi_{{{\text{Al}}_{2} {\text{O}}_{3} }}^{2} - 0.19\phi_{{{\text{Al}}_{2} {\text{O}}_{3} }} + 1), \hfill \\ \frac{{k_{hnf} }}{{k_{f} }} = (306\phi_{\text{Cu}}^{2} - 0.19\phi_{\text{Cu}} + 1)(306\phi_{{{\text{Al}}_{2} {\text{O}}_{3} }}^{2} - 0.19\phi_{{{\text{Al}}_{2} {\text{O}}_{3} }} + 1). \hfill \\ \end{gathered}$$

### Physical quantities

The physics parameters of interest are shown as.

13 $$Cf = \frac{{\tau_{w} }}{{\rho_{hnf} u_{e}^{2} }},\tau_{w} = \mu_{hnf} \left( {\frac{\partial u}{{\partial y}}} \right)_{y = 0} ,Nu_{x} = \frac{{xq_{w} }}{{k_{f} \left( {T_{w} - T_{0} } \right)}},q_{w} = - \left[ {k_{hnf} + \frac{{16\sigma^{*} T_{\infty }^{3} }}{{3k^{*} }}} \right]\left( {\frac{\partial T}{{\partial y}}} \right)_{y = 0} ,$$

The simplified form are.

14 $$R_{e}^{0.5} Cf = \left( {\frac{{\mu_{hnf} }}{{\mu_{f} }}} \right)f^{\prime\prime}(0),\,\,R_{e}^{ - 0.5} Nu_{x} = - \left( {\frac{{k_{hnf} }}{{k_{f} }} + \frac{4}{3}Rd} \right)\Theta^{\prime}\left( 0 \right).$$

## Entropy rate

Entropy refers to the thermal energy amount that is converted into waste heat under a process that indicates the thermal productivity of the whole system. By computing entropy generation the researchers focused on the waste heat production and optimize their structure to minimize it. This grows the system's performance and makes it suitable to control energy consumption and mainly focused on the lower cost as stated in^26–28^. In this research, the inclusion of the Entropy regime and Bejan number formation is mainly designed under the effect of the physical parameters for the optimization of energy. The relevant idea to the model problem is stated as.

15 $$\begin{aligned} S_{g} & = \frac{1}{{T_{\infty }^{2} }}\left( {k_{hnf} + \frac{{16\sigma^{*} T_{h}^{3} }}{{3k^{*} }}} \right)\left( {\frac{\partial T}{{\partial y}}} \right)^{2} + \frac{{\mu_{hnf} }}{{T_{\infty } }}\left( {\frac{\partial u}{{\partial y}}} \right)^{2} , \hfill \\ S_{G} & = \left( {\frac{{k_{hnf} }}{{k_{f} }} + Rd} \right)\lambda \left( {\Theta^{\prime}} \right)^{2} + Br\left[ {\left( {f^{\prime\prime}} \right)^{2} \left( {1 - \phi_{\text{Cu}} } \right)^{ - 2.5} \left( {1 - \phi_{{{\text{Al}}_{2} {\text{O}}_{3} }} } \right)^{ - 2.5} } \right]. \hfill \\ \end{aligned}$$

Here $\lambda = \frac{{T_{w} - T_{\infty } }}{{T_{\infty } }},S_{G} = \frac{{S_{g} T_{\infty } }}{{\left( {T_{w} - T_{\infty } } \right)}}$ are the difference in temperatures and entropy rate.

### Bejan number

The ratio of Entropy that happens due to heat transfer and total Entropy is called the Bejan number. The Bejan number provides an indoor dominant heat transfer mode in a system, with high values indicating the conduction dominance of low values indicating the dominance of convection. This information helps optimize the design of the solar collector, which focused on temperature control and heat transfer rates. This is how the Bejan number plays a key role in the deployment of an efficient and durable thermal system.

16 $$Be = \frac{{\frac{{k_{hnf} }}{{k_{f} }}\left( {\Theta^{\prime}} \right)^{2} \lambda }}{{\lambda \left( {Rd + \frac{{k_{hnf} }}{{k_{f} }}} \right)\left( {\Theta^{\prime}} \right)^{2} + Br\left[ {\left( {f^{\prime\prime}} \right)^{2} \left( {1 - \phi_{1} } \right)^{ - 2.5} \left( {1 - \phi_{2} } \right)^{ - 2.5} } \right]}}.$$

## Method of solution

**Figure 2:**
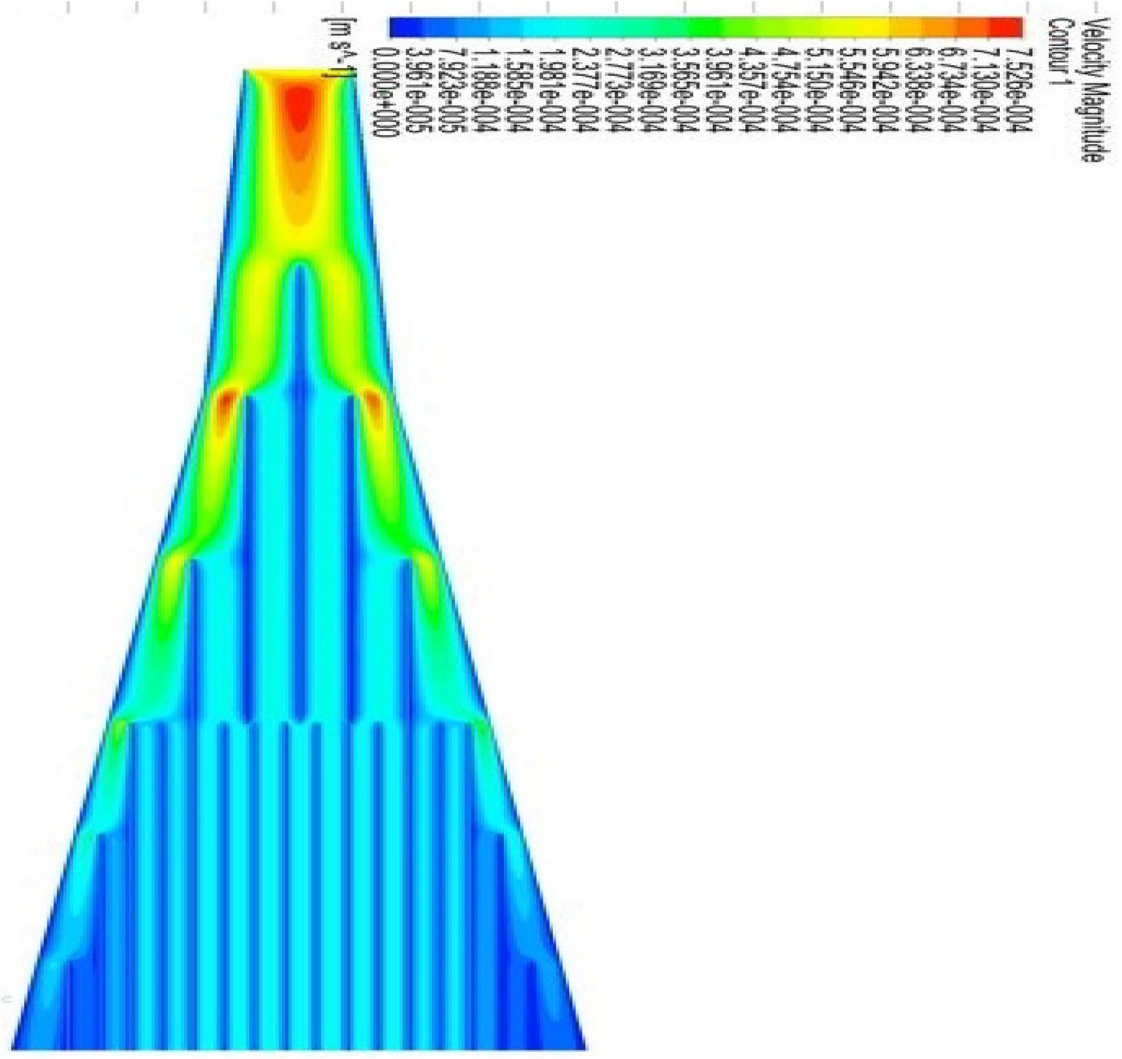
Simulation for the HNFs flow in terms of stagnation.

The problem of the transformation model expressed in the Eqs. (7, 8) is modified in the first-order system. Also, the physical conditions in Eq. (9) are set for the initial value problem. The well-known numerical technique (Runge–Kutta (RK-4)) referred to in^29,30^ is used to solve the problem. Machine learning software (Multi-Physics Simulation) is also used to validate the proposed model through the (CVFEM) approach^31–34^. This new technique deals with the Direct PDE solution. Besides, the high performance of the machine makes convergence more appropriate. Figure 1a illustrates the geometry of the proposed problem as a solar panel which is conceived from Fig. 1b in the form of Grids. Figure 1c is the flowchart of the suggested problem. The flow of hybrid nanofluids at the stagnant point in the presence of the Riga plate is simulated and shown in Fig. 2. The uniform parallel distribution of the hybrid nanofluid stream shown in the Figures depends on the parallel Riga plates. This uniform distribution is vital for optimizing the solar collector by optimizing the solar radiation. The flow distribution is progressively increased to maintain uniform fluid flow and reduce turbulence during stagnation.

## Results and discussion

**Figure 3:**
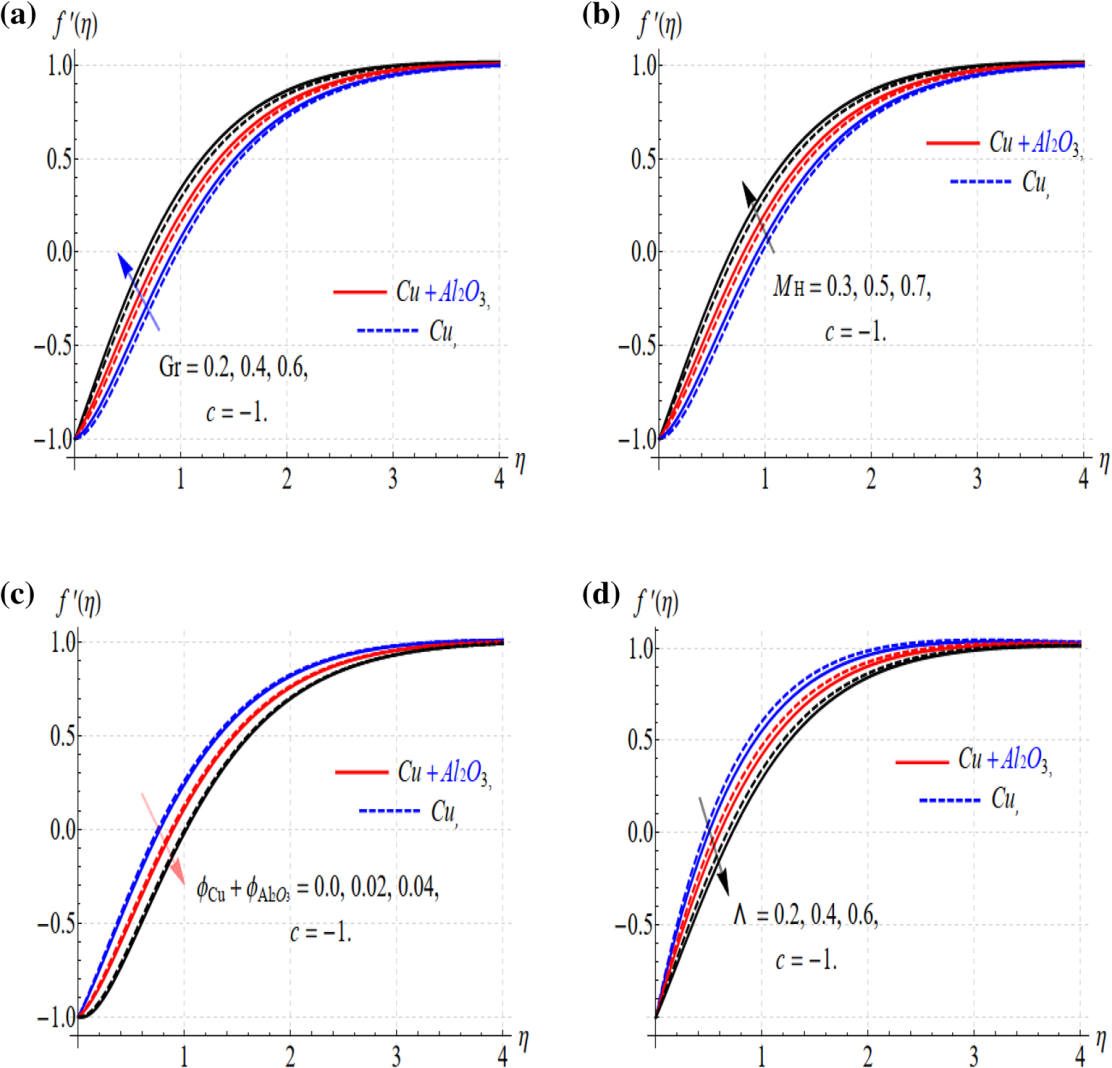
(**a**–**d**) Gr, M_H_, $\phi$ and $\Lambda$ versus $f^{\prime}(\eta )$ when c =  − 1.

**Figure 4:**
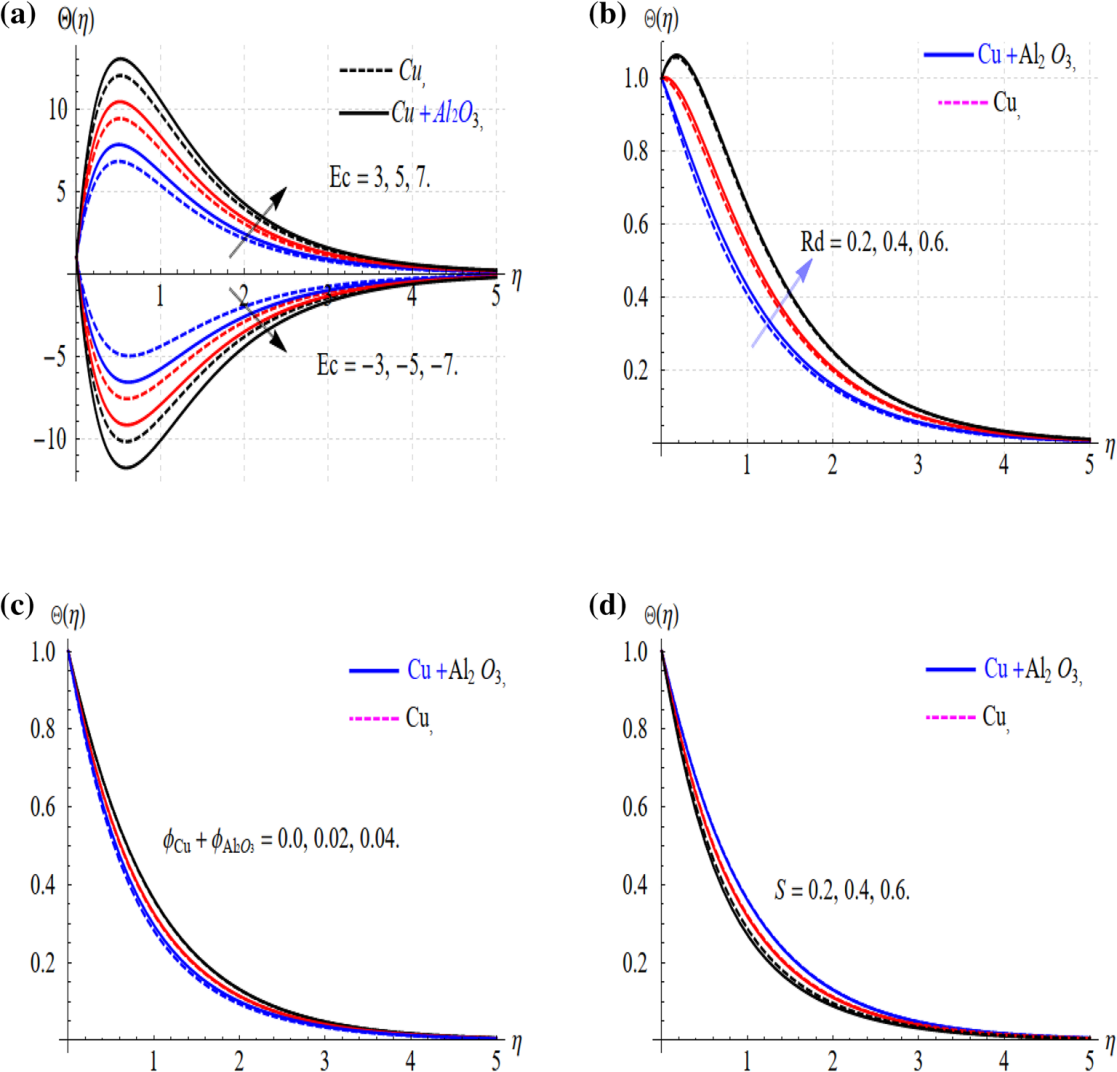
(**a**–**d**) Impact of Ec, Rd, $\phi$ and S on Θ(η).

**Figure 5:**
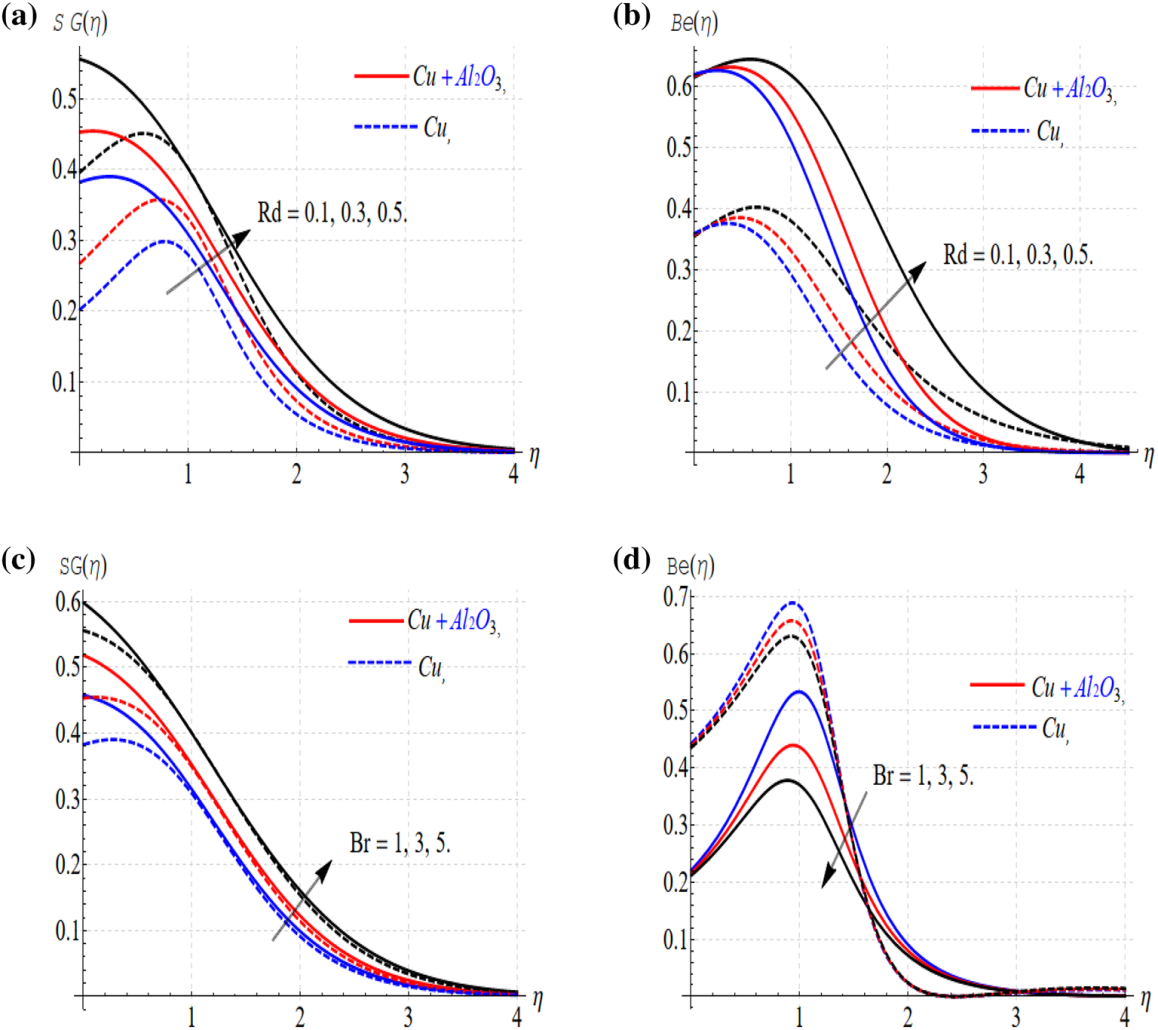
(**a**–**d**) Influence of the parameters versus S_G_ and Be.

**Figure 6:**
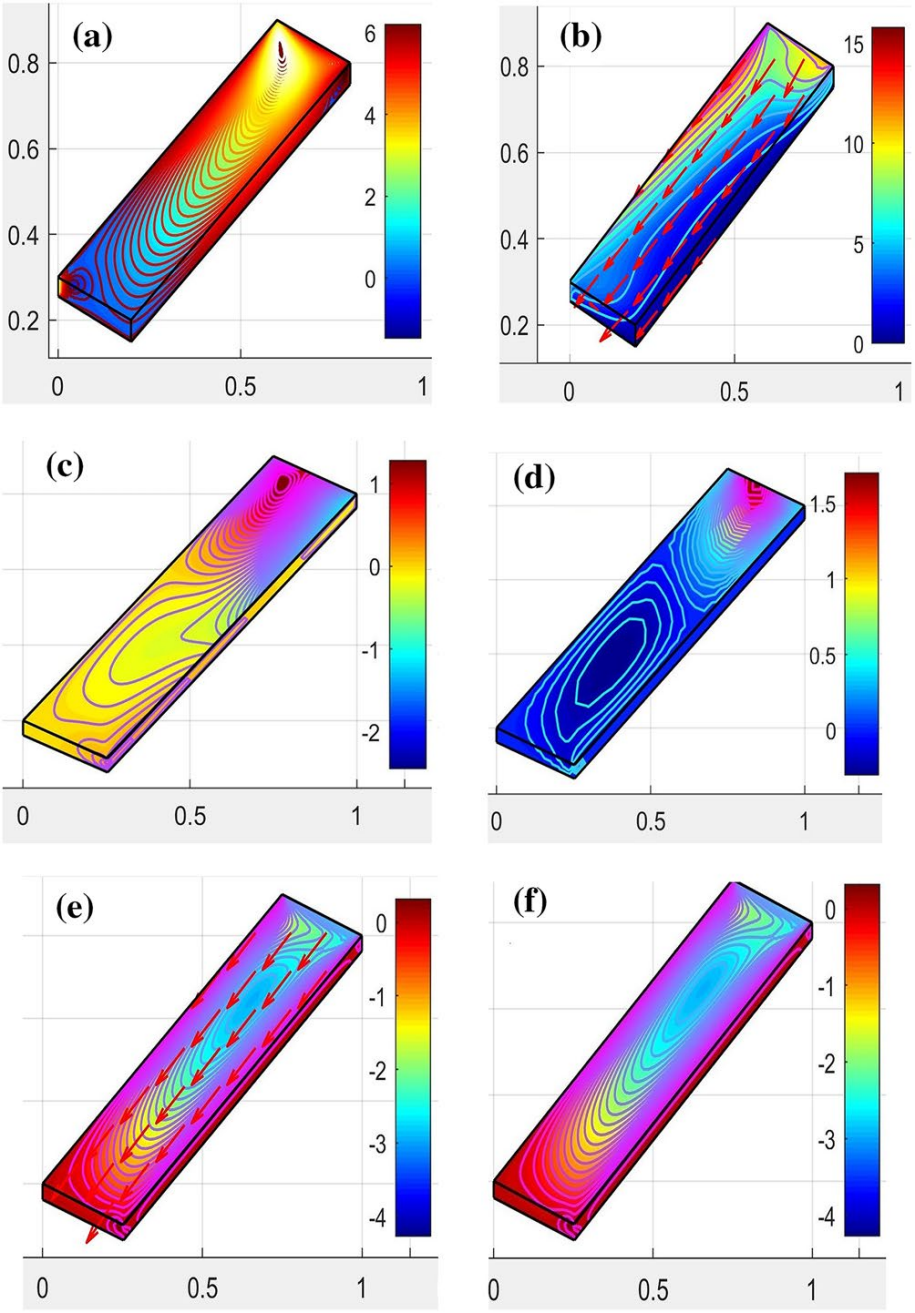
The solar panel, presentation for the fluid distribution in terms of the streamlines.

**Figure 7:**
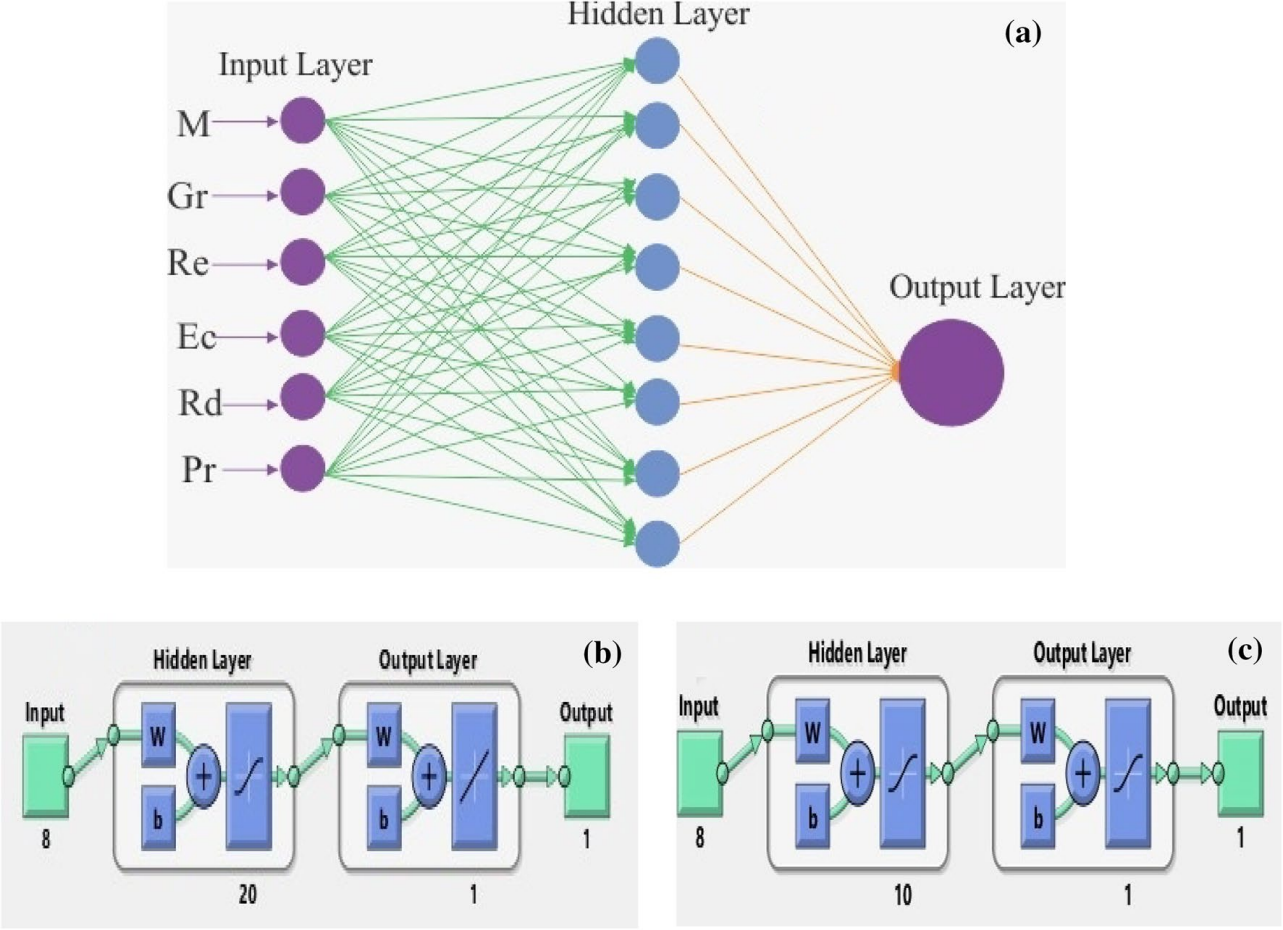
(**a**–**c**) Layers distribution for the model problem using the stagnation.

**Figure 8:**
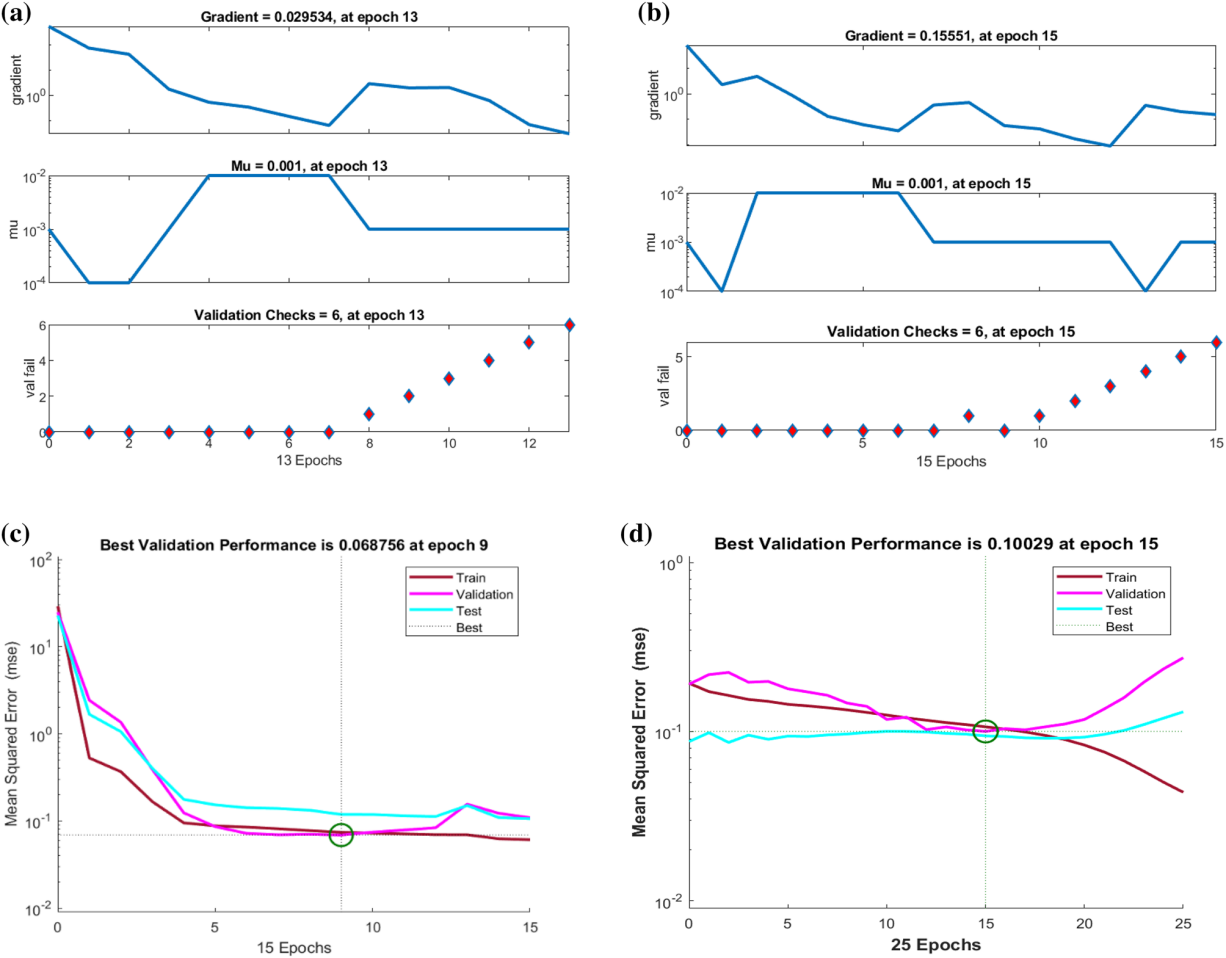
(**a**–**d**) Performances of the MSE and STs for the model.

**Figure 9:**
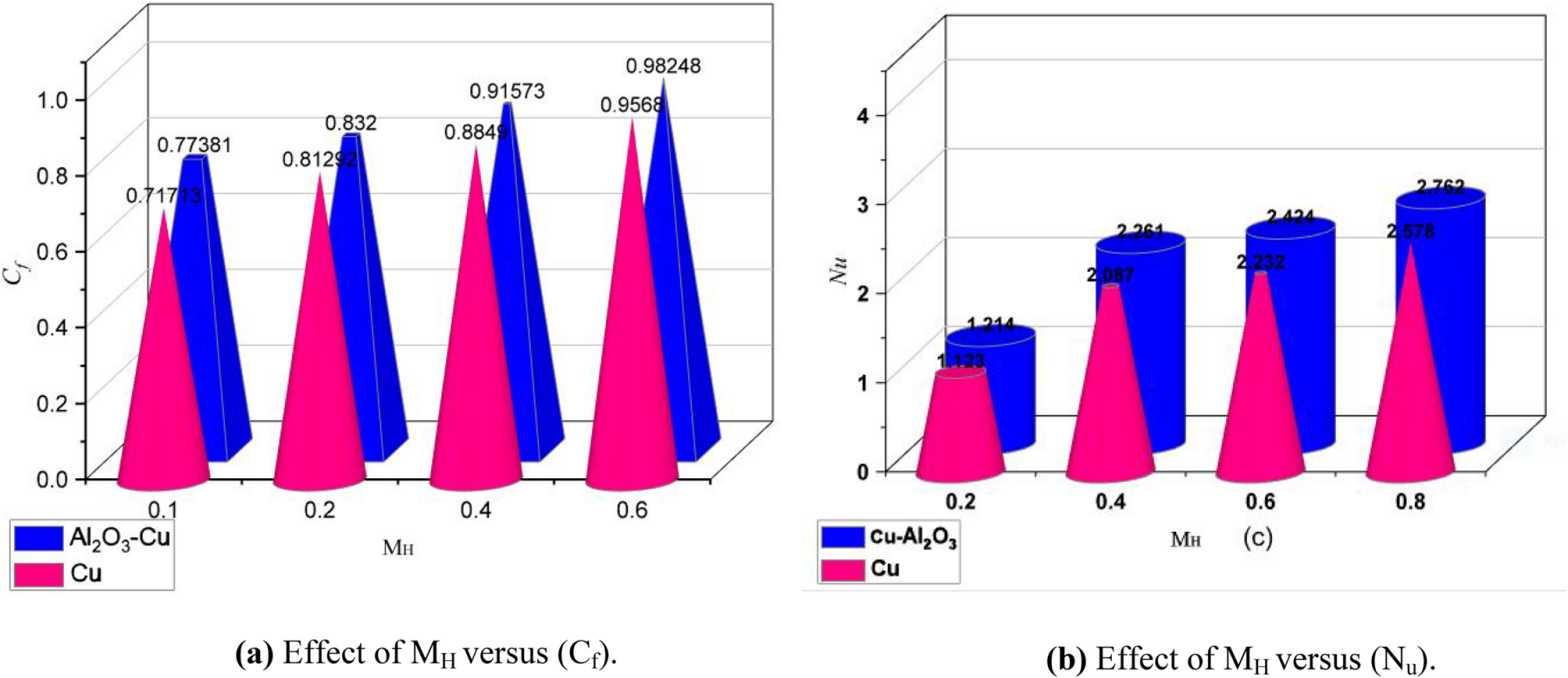
(Cf) and (Nu) subject to M_H_.

The parameters have an important role in the dimensionless form of the model problem and the effect of these parameters predicts the expected results. The flow field and thermal profile are investigated under the variation of the physical parameters and the co-relation of these results with the possible outputs are displayed and discussed. Parameters $\phi$, Gr, S, M_H_, Λ, Ec, and $Rd$ have expressive dynamic effects on the diffusivity. The flow velocity field ($f^{\prime}(\eta )$) and thermal distribution ($\Theta (\eta )$) are affected by the variations of these parameters. The Riga plate, stagnation point flow rate reduces the turbulence nature of the flow and thus provides a uniform distribution to the flow field. These important factors are to, optimize solar radiation. All these features are displayed in Figs. 3, 4, 5, 6, 7, 8, 9 and are also presented in numerical tables.

### Analysis of velocity and temperature profiles

The physical parameters (Gr, M_H_, $\phi$ and Λ) influence the hybrid nanofluids flow keeping (c < 0), have been displayed in Fig. 3a–d. The velocity field $f^{\prime}(\eta )$ upsurge with the augmentation of (Gr and M_H_) as shown in Fig. 3a and b. The enhancement in the velocity field occurs with the increase in the Grashof numbers which happens due to the natural convection. Also, an increase in the values of the Riga plate parameter (M_H_) increases the fluid motion. This is because effective flow control can only be obtained by applying the Lorentz force in a direction parallel to the wall. An external electric field generated by the Riga plate controls (HNFs) flow, resulting in the wall parallel Lorentz force. The response is quite progressive in the case of the hybrid nanofluids as compared to the (Cu) nanofluids. While this situation is quite different in the case of the $\phi$ and Λ as connived in Fig. 3c and d. Physically, this all happens with the generation of the resistive force which happens with the increasing values of these parameters. This parameter range is settled based on the convergence of the problem. Also, the uniform flow distribution is conceived in the proposed range of the embedded parameters. In addition, the graphs in Fig. 4 describe the consequences resulting from $\Theta (y)$ as a function of augmentations in Ec, Rd, $\phi_{\text{Cu}} + \phi_{{{\text{Al}}_{2} {\text{O}}_{3} }}$ and S, independently. Figure 4a provides an explicit illustration of Ec's influence on the dimensionless temperature in rising and decaying cases. The positive value of the viscous term (Ec > 0) improves the thermal field and this difference is quite dominant in the case of the hybrid nanofluids. This shows that hybrid nanofluids are operational in the heat transfer rate as related to the other traditional fluids. Similarly, the cooling effect is also prominent in the case of the negative values of the Eckert number (Ec < 0).

It happens due to the viscous interactions among the dissipation of the heat friction and fluid layer and as a result, the fluid temperature increases or decreases depending on the nature of (Ec). The solar radiation term (Rd) improves the thermal field for its larger values and this effect is quite impressive in the case of the (Cu + Al_2_O_3_) as displayed in Fig. 4b.

Nanoparticle volume fraction augmentation implied the enhancement in the thermal profile as shown in Fig. 4c. Again, the comparison shows that hybrid nanofluids are quite remarkable to improve the rate of thermal transport. The unsteady parameter (S) also, provides the augmentation to the temperature profile for its larger values as displayed in Fig. 4d.

Entropy generation (S_G_) and (Be) improve with the enlargement in solar radiations. As shown in Fig. 5a and b. The conduction of heat transposed to thermal radiation transport is the property of solar radiation. The radiation term provides more heat to the system and consequently improves thermal conductivity. In other words, an augmentation in (Rd) leads to an extra release of radiation, and consequently, the entropy generation rate is improved. This is because irreversible dominates the thermal conductivity over total irreversibility.

Figure 5c and d show that (S_G_) (*Βe)* varies with the Brinkman number. The internal temperature of the fluids boosts up with the increasing values of the Brinkman number and consequently enhancing the entropy. But this trend is in opposing action in the case of Bejan's issue. Again, the trends are fairly impressive when it comes to hybrid nanofluids. Also, these results show that irreversibility analysis optimizes the solar collector.

The range of these parameters described in Figs. 3 and 4 is adjusted for the proposed model and strong convergence. Moreover, these parameters are found in good agreement with the stagnation point flow and uniform distribution of the flow pattern. The hybrid distribution of the Al2O3-Cu nanofluids can be seen in Fig. 6a–d. This analysis has been done through (CVFEM), and the grid selection has been displayed in Fig. 1b. For this whole analysis, advanced software (FEA Tool Multiphysics) has been used. This software has the benefit that a built (CVFEM) tool already exists and just editing is required to settle the physical conditions and thermophysical properties of the hybrid materials. This built function also provides the stability, homogeneity, and validity of the model problem. The nonuniform flow distribution is shown in Fig. 6a,c,d, signifying that the panel is not auspicious for concentrated thermal energy gain. The streamlines show that the hybrid nanofluids flow is not uniformly distributed and not favorable to confirm the stability.

The essential agreement for uniform flow spreading and it is conceivable by simulations to adjust the panel according to the model proposed as revealed in Fig. 6b,e,f. In Fig. 6f, the distribution is quietly uniform as shown in Fig. 6e through directed lines which makes the flow distribution compatible. The impact of the important parameters of wall friction and the heat transfer rate is calculated and displayed in the form charts.

Some numerical results are performed from the proposed model to check the theoretical results using the MATLAB ode45 solver using the initial conditions as mentioned in^35^.

Figure 7a shows the multi-layer technique based on a single entry, layers hidden with 10 and 20 neurons as well as exit layers. Figure 7b and c show the neural network procedure, in which the algorithm-based solution is used for the optimization of Bayesian regulation, as well as MSE performance. The Epochs are chosen 500, times for performances, and execution, and Mu is also offered for the problem of the stagnation point flow model. Figure 7b and c indicate the DNN performance with 10 and 20 numbers of neurons in the hidden layers. The neural network procedure and Bayesian regularization method are used in combination which is found more effective as compared to other solvers. The Bayesian regulation tactic is more realistic to condense the required duration of cross-validation. This technique is known as a mathematical method that modifies the performance of non-linear regression in the system of special statistics by peak regression. The input domain and range are 0 and 1 including 0.01 step size. The mean square error (MSE) is displayed in Fig. 8a–d which predict the state transitions (STs) to handle the Stagnation point flow with the Riga plate.

The choice of epochs is chosen at 500 for the proposed model. These results strongly validate the proposed model.

The impact of the skin friction versus (M_H_) is displayed in Fig. 9a,b for the skin friction and Nusselt number. Both physical parameters are increases with the larger values of (M_H_). The increasing effect is quite effective in the case of hybrid nanofluids. The strengthening performance controls the thermal performance and improves the optimization. It should be noted that all of the above results are recorded with improvements for hybrid nanofluids since hybrid nanoparticles have more thermal responses than mono nanoparticles. Also, hybrid nanofluids tend to provide reasonable thermal performance to the base fluid.

**Figure 10:**
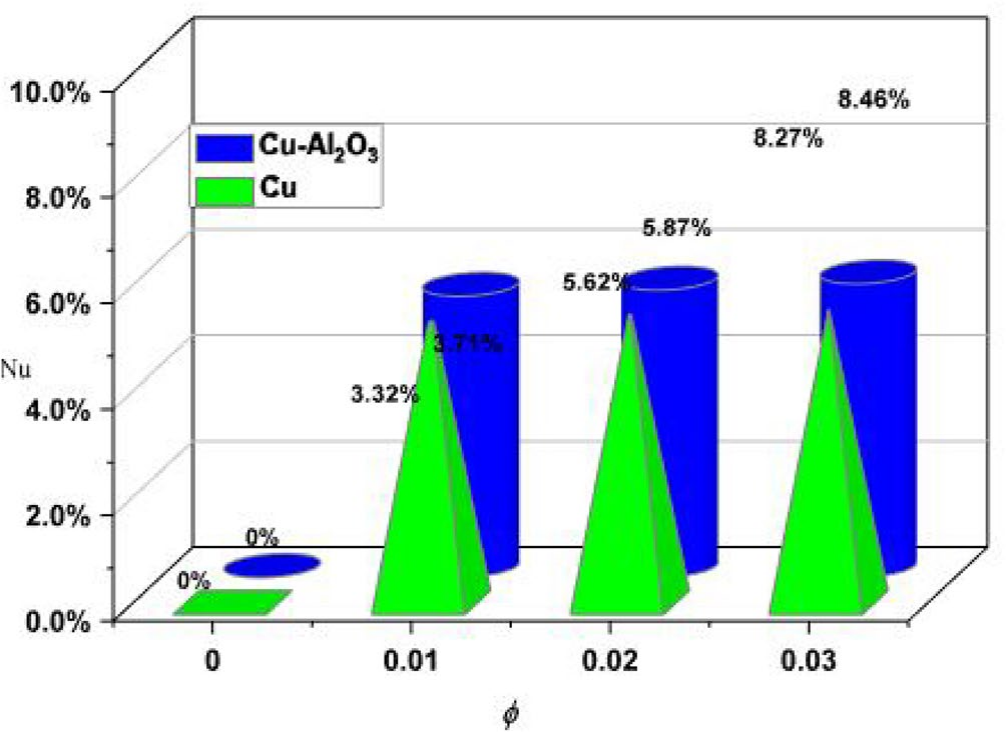
Thermal enhancement in % subject to $\phi$.

Figure 10 shows the percentage comparison of the volumetric fraction of nanoparticles and thermal transfer. It is observed that hybrid nanofluids (Cu-Al_2_O_3_) are more efficient than nano liquid (Cu). Model comparability is based on stability, convergence, and validation. Approval of this model is verified in two cases. One in terms of machine learning (neural network) and the other through comparisons with the available literature. The common parameter of acceleration parameter α = $\frac{a}{b}$ is used for the validation while the rest of the parameters are kept silent. The closed agreement of the present analysis and published analysis is calculated and displayed in Table 2. A closed agreement has been found which validates the present results.

**Table 2:** Validation of skin friction results in published work with common parameters.

	Wang^8^	Ishak et al.^36^	Lok and Pop^37^	[Present]
**α = **$\frac{{\varvec{a}}}{{\varvec{b}}}$	$f^{\prime\prime}(0)$	$f^{\prime\prime}(0)$	$f^{\prime\prime}(0)$	$f^{\prime\prime}(0)$
$-2$	0.2438231	0.2523143	0.2535231	0.2536231314
$-1.3$	0.432863294	0.442142286	0.4432165	0.44332176142
$-1.0$	1.483285531	1.5632184832	1.56465231218	1.5633521218
$-0.5$	1.623167412	1.72871235120	1.72073521421	1.720345197513

## Conclusion

The present model is based on fluid stagnation to optimize the solar collector. The Riga plate and entropy generation are added in combination to enhance the optimization process. The irreversibility also efficiently works to optimize the thermal profile. The solution of the proposed model is obtained using the numerical RK-4 method. The CVFEM approach is used to check the flow performance in a uniform pattern. Machine learning neural networking is applied to test the convergence of the proposed model and validate the obtained results. The main outcomes are presented as.

- It has been observed that irreversibility, stagnation, and Riga plates, are mutually very effective in the optimization of the solar collector.
- The percentage analysis approved that hybrid nanofluids are more prominent to expedite the heat transfer rate as compared to the simple fluids of nanofluids.
- Bayesian regularization is implemented to confirm the convergence of the problem and validate the obtained results.
- The simulation through high performance machine approved the validation of the model problem.
- Also, for the processes of high-rated thermal transfer, such as heat exchangers, (HNFs) can be the best option. Also, to achieve optimal flow rates, the performance of (HNFs) accompanied by an appropriate Riga setup is appreciated.

The current study is extendable in terms of the broad field, by adding the slip boundary conditions. The hall current, different nanomaterials, and ternary hybrid nanofluids study considering the recent model is also a new addition.

## Data Availability

Data are however available from the authors upon reasonable request from the corresponding authors.

## List of symbols

$u$,$v$: Terms of the velocity (m s^−1^)
$$j_{0}$$: Electrodes density
$u_{e}$: Velocity in terms of stagnation (m s^−1^)
$$Ec$$: Eckert number
$$M_{0}$$: Magnets
$$S$$: Unsteady term
$$MH$$: Hartmann number
$\Theta ,f$: Thermal and velocity profiles
$${\text{Re}}$$: Reynold number
$T$: Temperature of fluid $\left( {\text{K}} \right)$
$Gr$: Thermal Grashof number
$T_{w} ,T_{\infty }$: Wall and ambient temperatures $\left( {\text{K}} \right)$
$$g$$: Gravitational acceleration
$$\Pr$$: Prandtl number

## Greek symbols

$\mu_{hnf}$: Hybrid nanofluid viscosity $\left( {{\text{mPa}}} \right)$
$\eta$: Dimensionless transform variable
$Rd$: Solar radiation factor
$cp_{f}$: Specific heat of base liquid
$\alpha$: Stretching shrinking parameter
$$\beta_{T}$$: Thermal expansion
$$\Lambda$$: Dimensionless parameter
$$\sigma_{nf}$$: Electrical conductivity
$\phi$: Nanoparticle volume fraction
$cp_{hnf}$: Specific heat of hybrid nanofluid
$\rho_{f}$: Base fluid density (kg m^−3^)
$\mu_{f}$: Viscosity of the base fluid $\left( {{\text{mPa}}} \right)$
$$k_{hnf}$$: Thermal conductivity
$\rho_{hnf}$: Hybrid nanofluid density (kg m^–3^)
